# Rapid Targeted Method of Detecting Abused Piperazine Designer Drugs

**DOI:** 10.3390/jcm10245813

**Published:** 2021-12-12

**Authors:** Anna Welz, Marcin Koba, Piotr Kośliński, Joanna Siódmiak

**Affiliations:** 1 Department of Toxicology and Bromatology, Faculty of Pharmacy, Collegium Medicum Nicolaus Copernicus University, 85-089 Bydgoszcz, Poland; kobamar@cm.umk.pl (M.K.); piotr.koslinski@cm.umk.pl (P.K.); 2 Department of Laboratory Diagnostics, Faculty of Pharmacy, Collegium Medicum Nicolaus Copernicus University, 85-094 Bydgoszcz, Poland; asiapollak@wp.pl

**Keywords:** piperazine derivatives, benzylpiperazine derivatives, phenylpiperazine derivatives, designer drugs, hallucinogenic effects, LC-MS, stimulants

## Abstract

Piperazine derivatives belong to the popular psychostimulating compounds from the group of designer drugs. They are an alternative to illegal drugs such as ecstasy and amphetamines. They are being searched by consumers for recreational use due to their stimulating and hallucinogenic effects. Many NPS-related poisonings and deaths have been reported where piperazines have been found. However, a major problem is the potential lack of laboratory confirmation of the involvement of piperazine derivatives in the occurrence of poisoning. Although many methods have been published, piperazine derivatives are not always included in a routine analytical approach or targeted toxicological analysis. There is an increasing need to provide qualitative evidence for the presence of piperazine derivatives and to ensure reproducible quantification. This article describes a new rapid method of detecting piperazine derivatives in biological material, using LC-MS. All target analytes were separated in a 15 min run time and identified based on the precursor ion, at least two product ions, and the retention time. Stable isotopically labeled (SIL) internal standards: BZP-D7, mCPP-D8 and TFMPP-D4 were used for analysis, obtaining the highest level of confidence in the results. The proposed detection method provides the analytical confirmation of poisoning with piperazine designer drugs.

## 1. Introduction

Available literature data indicates a growing popularity of new psychoactive substances (NPS) [1]. These products, also known as designer drugs, were introduced to the market as an alternative to illicit drugs in order to circumvent legal sanctions [2,3]. The increasing number of NPS, easy accessibility via the Internet, and the growing number of consumers, has created a complex drug problem in Europe [1,4].

**Figure 1:**
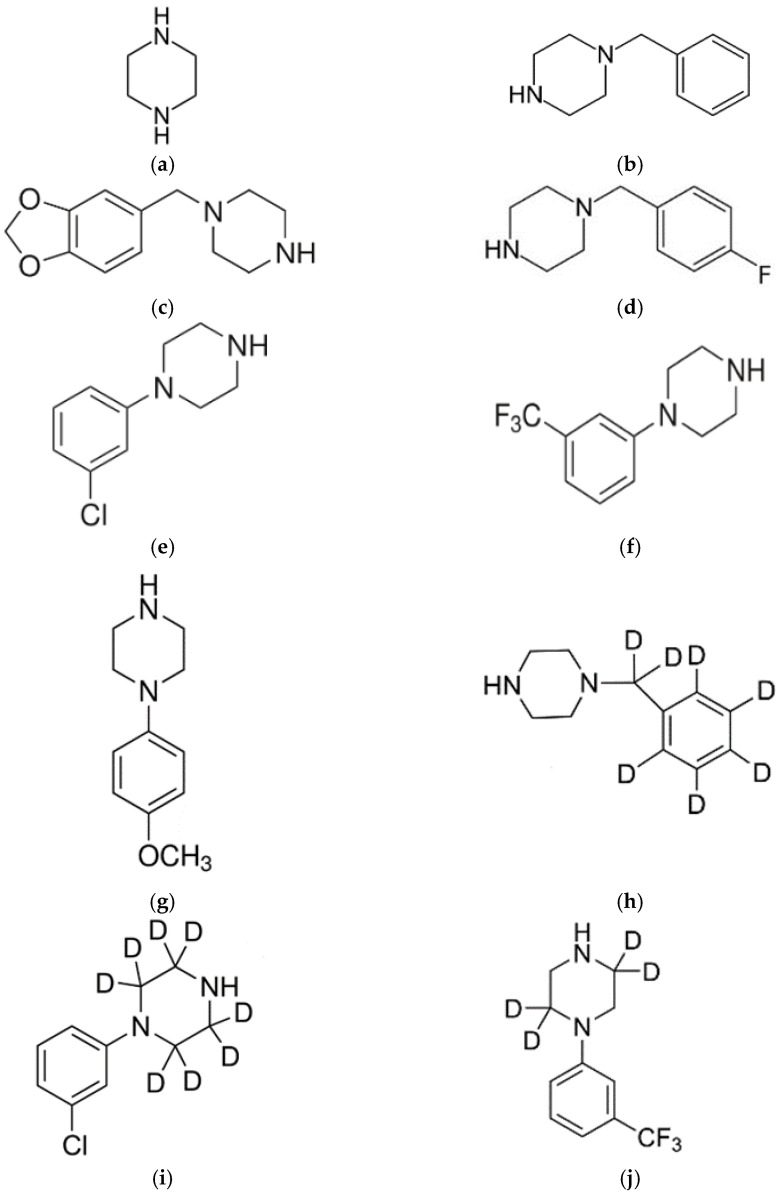
Chemical structures of (**a**) piperazine, piperazine derivatives: (**b**) *N*-benzylpiperazine (BZP), (**c**) 1-(3,4-methylenedioxybenzyl)piperazine (MDBP), (**d**) 1-(4-fluorobenzyl)piperazine (pFBP), (**e**) 1-(3-chlorophenyl)piperazine (mCPP), (**f**) 1-(3-trifluoromethylphenyl)piperazine (TFMPP), (**g**) 1-(4-methoxyphenyl)piperazine (MeOPP) and deuterated internal standards (**h**) BZP-D7, (**i**) mCPP-D8, (**j**) TFMPP-D4.

Piperazine derivatives are popular compounds with stimulating and hallucinogenic effects similar to MDMA [5,6,7,8,9]. In terms of chemical structure, they are derived from piperazine, a cyclic molecule with two nitrogens in the opposite position and four carbon atoms occurring between the two nitrogen atoms [6,10,11]. Piperazine derivatives present in designer drugs can be divided into two groups: benzylpiperazines, e.g., *N*-benzylpiperazine (BZP), 1-(3,4-methylenedioxybenzyl)piperazine (MDBP), 1-(4-fluorobenzyl)piperazine (pFBP), and phenylpiperazines, e.g., 1-(3-trifluoromethylphenyl)piperazine (TFMPP), 1-(3-chlorophenyl)piperazine (mCPP), 1-(4-methoxyphenyl)piperazine (MeOPP). Figure 1 shows the chemical structures of piperazine, the most popular piperazine derivatives occurring in designer drugs and deuterated internal standards used in this study. The products containing piperazine derivatives are most often sold as party pills, powders, capsules, tablets, liquid mixtures, injections and smoking forms [5,6,7,8]. The most common names of preparations containing various piperazine derivatives are: “Legal X”, “Legal E”, “Bliss”, “A2”, “Party pills”, “X4”, “Rapture”, “Lab-X”, “Cherries”, “Clear Light”,” Combo”, “The Good Stuff”, “Exodus” and “Herbal Ecstasy” [6,8,12,13].

BZP doses taken by consumers range from 50 to 250 mg and even up to 1000 mg [7,14,15], with the possibility of detection in the blood up to 30 h after ingestion [16]. TFMPP doses have been reported in the range of 5 to 100 mg [5,15,17]. The effect is not immediate, but it lasts from 6 to 8 h [5]. In turn, in the combination of BZP and TFMPP, the following proportions were recorded: 100 mg BZP and 30 mg or 50 mg TFMPP and 80 mg BZP and 40 mg TFMPP [12,18]. The reported mCPP content in a tablet sold as ecstasy was 45.8 mg [19]. On the other hand, doses of 0.25–0.75 mg/kg were tested in clinical trials with the use of mCPP [20,21]. For MDBP, the doses used by potential users have been around 50–100 mg [22].

Piperazine derivatives in designer drugs very often occur together with other psychoactive substances, for example, ecstasy, cocaine, amphetamine, ketamine and cannabis [7,8,23]. They are also often included in tablets sold to consumers as ecstasy or amphetamines. The most common in such products is a combination of BZP and TFMPP [8,12,23].

In studies of the pharmacological profile, piperazine derivatives were assessed as compounds leading to an increase in the level of dopamine (DA), serotonin (5-HT) and norepinephrine (NA) [5]. Increased levels of neurotransmitters can cause desirable as well as various adverse behavioral and clinical effects for patients [24,25]. Increased dopamine levels are associated with behavioral-stimulating effects, higher levels of norepinephrine may have an adverse effect on the cardiovascular system, and increased serotonin levels may cause entactogenic effects and, additionally, life-threatening serotonin syndrome [24,26]. It was also noted that each of the mood-changing compounds used has addiction potential [27].

Piperazine derivatives are typically sought by recreational users for their stimulating and hallucinogenic effects, extraordinary perception and experiences after ingestion [5,6,9,12,16,28]. Several studies have been carried out to explain the hallucinogenic effects of piperazine derivatives. Orsolini et al. [29] noted that the hallucinogenic properties of the tested NPS are the result of binding to the 5-HT2A receptor [29]. BZP binds to 5-HT1A-D and 5-HT2A-C receptors at the micromolar level [24]. It has been shown that BZP in high doses binds to the 5-HT2 receptor causing a mild hallucinogenic effect about 10 times less potent than MDMA [7,30,31]. Additionally, BZP inhibits DA reuptake and stimulates NA release for amphetamine-like effects [32]. Among the piperazine derivatives, TFMPP and mCPP bind the most strongly to serotonin 5-HT1A-D and 5-HT2A-C receptors [24]. The TFMPP has more direct serotonergic activity showing selective binding to 5-HT1 and 5-HT2 [32]. Binding to the 5-HT2A receptor may lead to changes in perception and cognitive functions [3]. Additionally, it has been shown that the action of mCPP as an agonist 5-HT2C receptors is associated with decreased appetite and activation of 5-HT3 receptors may be the cause of nausea [6,33]. The combination of BZP with TFMPP or mCPP shows the ecstasy profile because BZP affects DA release and TFMPP and mCPP are direct and indirect serotonergic agonists and affect serotonin release [34].

Consumers of piperazine derivatives claim that the effects experienced with BZP and TFMPP are similar to those of amphetamines, but without some of the unpleasant side effects, and mistakenly believe it is a “safe alternative” to MDMA [12]. Over the past few years, a multidirectional assessment of the cytotoxic activity of piperazine designer drugs has been carried out. The research of Dias da Silva et al. [12] proved that BZP-TFMPP mixtures, depending on the combination ratio, were much more hepatotoxic than MDMA and amphetamines [12]. Hepatic detrimental effects have also been shown depending on the type of piperazine derivative and its concentration [35]. Arbo et al. [36] justified the cardiotoxic effect of piperazine derivatives at the cellular level [36]. The authors noted that all tested compounds caused concentration-dependent cytotoxic effects, among which TFMPP showed the strongest effect. Additionally, cardiotoxicity may be the result of an increased concentration of noradrenaline in the blood [37]. Persona et al. [38] presented the effect of BZP on the induction of the mitochondrial apoptosis pathway in glial cells of nervous tissue [38]. Another study showed a negative effect of BZP and TFMPP on sensory processing such as attention, memory updating and auditory information processing [18]. Zwartsen et al. [39] in their studies showed an inhibitory effect on the neuronal activity by piperazine derivatives depending on their concentration [39].

In order to confirm the participation of piperazine derivatives in the occurrence of poisoning, information provided by clinicians about the symptoms of poisoning is necessary [11]. Some symptoms may be specific to piperazine derivatives. Typically, there is tachycardia, an increase in systolic and diastolic blood pressure, and a very characteristic pupil dilation [5,7,23,31,40]. Convulsions, bruxism, agitation, dissociative symptoms and fever are also observed [5,6,7,8,23,31,32,41]. Confirmation of the participation of piperazine derivatives in the occurrence of poisoning is necessary for comprehensive diagnosis and therapy of patients [11]. This is all the more important as the concentrations in serum or urine may not closely correlate with the observed clinical effects of poisoning [5,41].

Many NPS-related poisonings and deaths have been reported in the past where piperazine derivatives have been found [6,19,23,27,31,42]. However, each time a major problem is the lack of laboratory confirmation of the involvement of piperazine derivatives in the occurrence of poisoning [31]. Also, the underestimation of the number of poisonings may result from the lack of comprehensive analytical approaches to the detection and quantification of piperazine derivatives in biological samples [43]. So far, various methods of NPS detection using liquid chromatography (LC) or gas chromatography (GC) combined with mass spectrometry (MS) have been proposed [8,9,28,42,43,44,45,46,47,48]. GC-MS is the technique of choice for systematic toxicological analysis (STA), however it is necessary to use derivatization in sample preparation for piperazine derivatives [8,48]. HPLC-DAD procedures allow the detection of more polar and non-volatile compounds in the GC, but although repeatability is high unfortunately separation power and specificity are still inferior [8]. LC-MS is becoming an increasingly common apparatus, piperazine derivatives are not included in the routine analytical approach or targeted toxicological analysis yet [31]. For this reason, there is an urgent need to develop simple, fast and targeted methods for the detection of piperazine derivatives [9,43]. Rapid diagnosis is also needed because of frequent comorbidities, especially in times of pandemic [49].

According to the latest European Drug Report [50] piperazine derivatives are monitored substances that are harmful to health [50]. In addition, it has been noticed that the number of MDMA laboratories closed by law enforcement agencies is increasing in the European Union. It can be assumed that the potential users of piperazine derivatives are the same people who are taking ecstasy [13]. Limited access to MDMA products may increase the use of piperazine derivatives. Additionally, routine immunoassays target known drugs of abuse and do not detect piperazine derivatives [5,41,51]. The lack of an appropriate immunoassay indicates the need for an alternative technique to detect such compounds [51]. Therefore, there is an increasing need to provide qualitative evidence for the presence of piperazine derivatives and to ensure reproducible quantification.

The article describes a new rapid and targeted method of detecting of piperazine derivatives in biological material using LC-MS. In the process of validation of the presented method, the following parameters were assessed: linearity, measuring range, limit of detection (LOD), limit of quantification (LOQ), method repeatability and the use of stable isotopically labelled (SIL) internal standards. The method was found to be selective for all tested analytes, including extracts from serum and urine samples. All target analytes were separated in a 15 min run time including column equilibration. The sensitivity of the multiple reactions monitoring (MRM) was optimized for each compound to determine the highest intensity product ions. At least two MRM transitions were selected for each compound in order to obtain the highest selectivity and reproducibility of the method. All tested piperazine derivatives can be identified by their precursor ion, at least two product ions, and the retention time. The use of deuterated analogues: BZP-D7, mCPP-D8, TFMPP-D4 as preferred internal standards was documented, obtaining the highest level of confidence in the results. The proposed detection method provides strong analytical confirmation of poisoning with piperazine designer drugs and ensures repeatable quantitative assessment.

## 2. Materials and Methods

### 2.1. Reagents and Solvents

Standards of 1-benzylpiperazine dihydrochloride (BZP), 1-(3-chlorophenyl)piperazine hydrochloride (mCPP), 1-(3-trifluoromethylphenyl)piperazine hydrochloride (TFMPP), 1-(3,4-methylenedioxybenzyl)piperazine (MDBP), 1-(4-fluorobenzyl)piperazine (pFBP) and deuterated internal standards including BZP-D7, mCPP-D8 and TFMPP-D4 were purchased from Sigma-Aldrich company (Darmstadt, Germany). BZP, mCPP and TFMPP was received as a 1 mg/mL standard in methanol. MDBP and pFBP were received from of 10 mg powder. BZP-D7, mCPP-D8 and TFMPP-D4 was received as standards 100 μg/mL in methanol. Methanol hypergrade for LC-MS, acetonitrile hypergrade for LC-MS and Formic acid for LC-MS came from Sigma-Aldrich (Merck, Darmstadt, Germany). Sodium hydroxide was purchased from Avantor Performance Materials Poland S.A. (formerly POCH). Fresh water was obtained from a water filtration system from the demineralizer HLP 5UV Hydrolab (Straszyn, Poland). Biological samples intended for fortification (serum, urine) were collected from healthy volunteers after obtaining their informed consent (after obtaining the consent of the Bioethics Committee to conduct the study).

### 2.2. Instrumentation and Chromatographic Conditions

All analysis of piperazine derivatives was performed using an LCMS-8045 triple quadrupole liquid chromatograph mass spectrometer (LC-MS) equipped with a heated ESI probe with LabSolutions software. The components of the Shimadzu Nexera XR series HPLC system were the following: LC-20ADXR liquid chromatograph pump, SIL-20ACXR autosampler, DGU-20A3R degassing unit, LCMS-8045 liquid chromatograph mass spectrometer and CTO-20AC prominence column oven. Chromatographic separation was carried out on a Synergi 4 μm, Hydro—RP, 80A, C18 with polar endcapping, 150 × 2.00 mm LC column (Phenomenex, Inc. Torrance, CA, USA), in reversed-phase mode, with a mobile phase gradient. The components of the mobile phase were: water with 0.1% formic acid (mobile phase A) and LC-grade methanol with 0.1% formic acid (mobile phase B). The flow rate of the mobile phase was set to 0.5 mL/min in which starting condition was 10% mobile phase B. After 2 min of 10% B isocratic flow, the linear gradient program started at 10% B and increasing to 100% B for 8 min, followed by 1 min equilibration to 10% B and 4 min of isocratic flow. The total analysis time was 15 min and the column oven temperature was maintained at 30 °C. An autosampler was used to inject the sample and the volume of injection was 5 μL. The analyses used electrospray ionization in the positive mode. The DL temperature was 250 °C, the heater block temperature was 400 °C, the nebulizing gas flow was 3 L/min, the drying gas flow was 10 L/min and heating gas flow was 10 L/min. The use of a dynamic multiple reaction monitoring mode provided further optimization of the sensitivity, reproducibility and precision. At least two MRM transitions were selected for each compound.

### 2.3. Preparation of Samples for Calibration

Stock solutions of pFBP and MDBP were prepared by dissolving 1 mg of each powder in 1 mL of methanol. All dilutions of BZP, MDBP, pFBP, mCPP and TFMPP stock solutions were prepared by serial dilution with methanol. An internal standard stock solution was prepared by dilution with methanol to a final concentration of 100 ng/mL by combining BZP-D7, mCPP-D8 and TFMPP-D4. In order to check the linearity of the method, standard concentrations of piperazine derivatives were prepared: 1, 5, 10, 25, 100, 250, 500, 750 and 1000 ng/mL. All piperazine derivatives at the appropriate test concentration were combined into one sample and the internal standard was added by combining BZP-D7, mCPP-D8 and TFMPP-D4 each at 100 ng/mL, respectively. Samples were analysed in quadruplicate.

### 2.4. Preparation of Biological Samples

Serum and urine samples were prepared in individual portions of 100 μL. Piperazine derivatives and internal standards were added at a concentration of 100 ng/mL, respectively. The samples were then alkalized with 1 M NaOH or 3 M NaOH. Cold acetonitrile was added, samples vortexed and centrifuged for 5 min at 10.0 rpm. The obtained supernatants were filtered through a PES (polyethersulfone) membrane filters (ø = 25 mm, 0.45 µm pore size) into a final vials for measurement. Quantification of piperazine derivatives in all samples was performed using an LCMS-8045 triple quadrupole liquid chromatograph mass spectrometer (LC-MS) equipped with a heated ESI probe.

### 2.5. Validation of the Method

The validation process of the presented method was used to evaluate such parameters as: linearity, measuring range, limit of detection (LOD), limit of quantification (LOQ), repeatability of the method and the use of stable isotopically labelled (SIL) internal standards.

### 2.6. Linearity of the Method

The linearity range of the presented method was determined on the basis of a calibration curve obtained for each compound of the piperazine derivatives (in the measuring range from 0.001 to 1 μg/mL). The following calibration levels were adopted: 1 ng/mL, 5 ng/mL, 10 ng/mL, 25 ng/mL, 100 ng/mL, 250 ng/mL, 500 ng/mL, 750 ng/mL and 1000 ng/mL. Internal standards were added to each calibration sample at a concentration of 100 ng/mL. On the basis of the results obtained, the dependence of the analyte concentration as a function of the ratio of the analyte peak area and the internal standard was plotted. The data obtained were analyzed with the use of Microsoft Excel. From the obtained results, the regression equations for individual analytes and the coefficient of linear determination R^2^ were determined.

### 2.7. Analytical Limits: Limit of Detection (LOD) and Limit of Quantification (LOQ)

Assays were performed for six concentrations of analytes at a level close to the expected detection limit. From the obtained data, the value of the standard deviation was calculated, on the basis of which the curve s = f (c) was drawn (s-standard deviation, c-concentration). The limit of detection was calculated according to the formula: LOD = 3 s. The LOQ value, i.e., the lowest concentration of the substance that can be determined, was calculated on the basis of the equation LOQ = 3 LOD.

### 2.8. Repeatability of the Method

In order to assess the reproducibility of the method, control samples were determined at three known concentration levels of piperazine derivatives in the linear range of the method. The reproducibility of the retention times and surface areas of the piperazine derivatives was assessed during the day and between days.

### 2.9. The Stable Isotopically Labelled (SIL) Internal Standards

Three deuterated internal standards were used for each tested compound of piperazine derivatives: BZP-D7, mCPP-D8 and TFMPP-D4. The use of deuterated analogs as the best internal standards has been documented to obtain the most reliable results from the analysis of biological material.

## 3. Results

**Figure 2:**
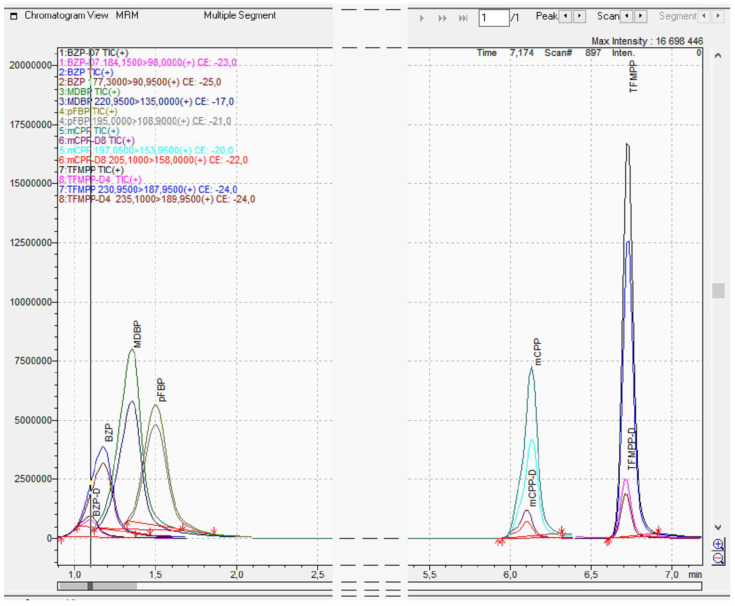
Liquid chromatography mass spectrometry (LC-MS) chromatogram (intensity vs. retention time) of piperazine derivatives and deuterated internal standards.

While developing a method for the detection of piperazine derivatives, individual elements of the chromatographic system were tested. Good chromatographic separation of piperazine derivatives in a relatively short time of analysis was obtained using a Synergi C18 column. Various stationary phases as well as the composition and proportions of mobile phase components during elution were tested. The chromatography was optimized by a gradient of eluents starting from a low concentration of organic solvent (10%), obtaining a good peak shape, high signal-to-noise ratio and good separation of analytes. The method has a total run time of 15 min including column equilibration. All tested piperazine derivatives can be identified by their different MRMs or retention times. An exemplary LC-MS chromatogram (intensity vs. retention time) of piperazine derivatives is shown in Figure 2. The sensitivity of the MRM was optimized for each compound to determine the highest intensity product ions as shown in Table 1. At least two MRM transitions were selected for each compound in order to obtain the highest selectivity and reproducibility of the method. The internal standard was added by combining BZP-D7, mCPP-D8 and TFMPP-D4 each at 100 ng/mL, respectively. Thanks to the combination used in this way, each internal standard was tested for each piperazine derivative.

**Figure 3:**
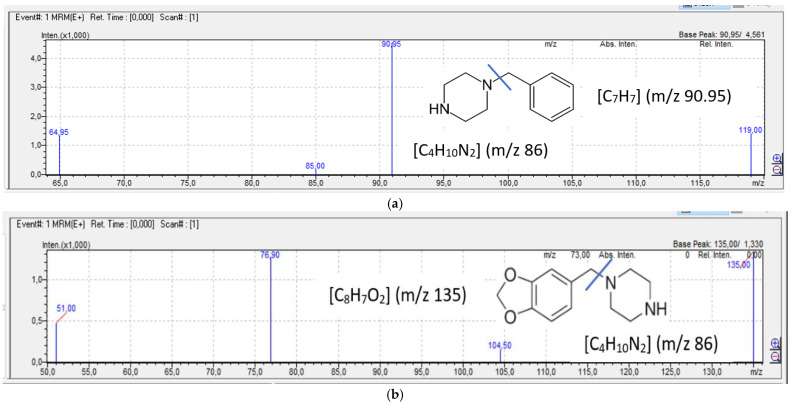

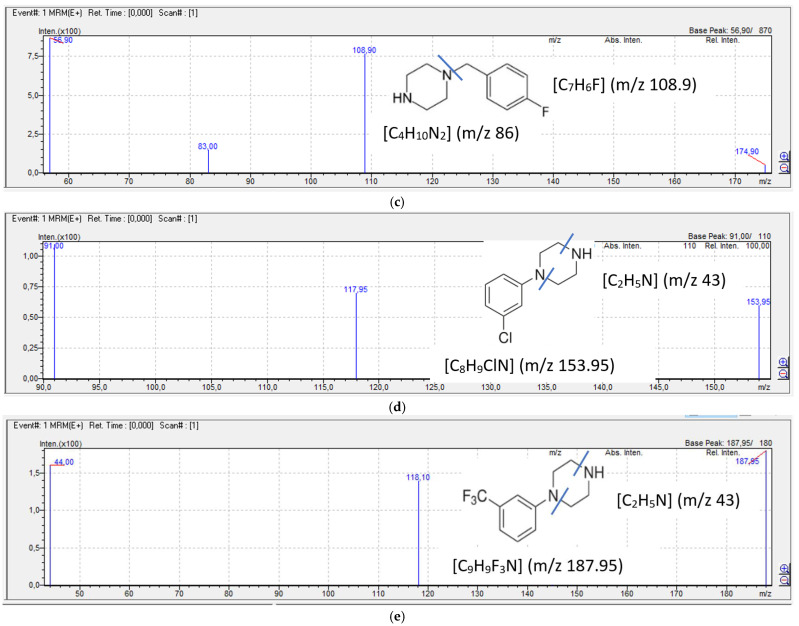
Mass spectra and major fragmentation patterns of piperazine designer drugs observed in mass spectrometry: (**a**) BZP, (**b**) MDBP, (**c**) pFBP, (**d**) mCPP, (**e**) TFMPP.

Observing the main fragmentation pattern, it can be concluded that in the case of benzylpiperazine derivatives, a constant neutral loss is produced, *m*/*z* 86, and in the case of phenylpiperazine derivatives, the neutral loss is *m*/*z* 43. Figure 3 shows the mass spectra and major fragmentation patterns of piperazine designer drugs observed in mass spectrometry.

### Validation of the Method (Linearity, Measuring Range, Limit of Detection (LOD), Limit of Quantification (LOQ) and Repeatability)

The method was found to be selective for all tested analytes, including extracts from serum and urine samples. The method was verified by the analysis of a 9-point calibration curve (*n* = 9). For each compound, linearity was confirmed over the calibration range with determination coefficient values varying between 0.990 and 0.999. The regression equation for individual analytes was determined from the results obtained. These results are presented in Table 2 together with the limit of detection and the limit of quantification. The obtained values of LOD and LOQ indicate the possibility of detecting very low concentration of piperazine derivatives at the level of single ng and event amount at the level of pg.

In order to assess the reproducibility of the method, the control samples (quality control, QC) were determined at three known concentration levels of piperazine derivatives in the linear range of the method (LQC, MQC, HQC). Based on the measurements of the retention times and peak areas, the coefficient of variation during the day and between days was calculated. The results obtained are presented in Table 3. The verified results are in line with the SWGTOX method validation guidelines [52].

The developed method was used for the identification and quantification of piperazine derivatives in fortified biological samples. Table 4 shows the results obtained from the analysis of biological material (urine, serum). Entries in “bold” highlight cases when deuterated analogues were used: BZP-D7, mCPP-D8, TFMPP-D4 as preferred internal standards.

## 4. Discussion

A method for the detection of piperazine derivatives in biological material using LC-MS was developed. The sensitivity of the MRM was optimized for each compound to determine the highest intensity product ions. At least two MRM transitions were selected for each compound in order to obtain the highest selectivity and reproducibility of the method. The results obtained were consistent with the majority of published literature data, which are presented in Table 5. This approach, involving the determination of two or even three product ions, also allows the elimination of natural or synthetic interfering substances found in biofluids [51]. An example is cotinine, a metabolite of nicotine which is often found in urine. Cotinine and BZP have the same starting mass and precursor ion, but cotinine does not have an equivalent product ion at *m*/*z* 91 avoiding possible misidentification of BZP use.

### 4.1. Repeatability of the Method

An extremely important stage in the development of the method was the selection of an internal standard. In previous literature reports, various internal standards were used in the analysis of piperazine derivatives, e.g., amphetamine-D5 for BZP [44,46], for mCPP, tramadol-D3 was used [43], mephedrone-D3 [46], DMPP [44], for TFMPP, phencyclidine-D5 was used [46], tilidine-D6 [43], DMPP (1-(3,4-dimethylphenyl)piperazine) [44] and amphetamine-D5 was used for MDBP [44]. It has been shown, however, that the greater the difference in the chemical properties of the internal standard and the tested compound, the greater the probability of inaccuracy in the bioanalytical method [53]. According to many scientific reports, the use of stable isotope-labeled (SIL) analogues as internal standards is the most recommended [51,53,54,55,56]. A molecule of stable isotope-labeled analyte analogue, co-eluting with the analyte and having the same physicochemical properties, should standardize the error caused by matrix effects [56].

In the presented method, three deuterated internal standards: BZP-D7, mCPP-D8 and TFMPP-D4 were used for each tested compound of piperazine derivatives. Targeted analyses indicate which of the applied internal standards should be used. The highest level of confidence in the results was obtained by using deuterated analogues as internal standards. The calculated ratios of the analyte response to the internal standard response were used to generate the calibration function and calculate the test sample concentrations. The co-elution process was observed for piperazine derivatives with a suitable deuterated analog, as follow: BZP and BZP-D7, mCPP and mCPP-D8, TFMPP and TFMPP-D4. On the other hand, there were minimal differences in retention times when using the deuterated BZP-D7 standard for MDBP and pFBP, and larger differences were noted when considering specific compounds and deuterated standards differing in structure (Table 1). Some analyses of piperazine derivatives in biological matrices showed lower recovery of the deuterated internal standard. Earlier studies have noted that deuterium-labeled compounds can cause unexpected recovery problems compared to analyte [54,56]. The reason for this effect may be differences in the physicochemical properties of the compound or the phenomenon of substitution of deuterium with free protons in mobile phases causing a drop in the IS signal [54,56]. This phenomenon may be important in the case of positive ionization, because acidification of mobile phases is a practice used to increase the signal [56]. Also, the target analyte may compete with the ionization of the internal standard, thus causing ion suppression or enhancement. Loss of deuterium can also be due to traces of water usually present in acetonitrile [54]. Moreover, a slight difference in lipophilicity between the original compounds and their deuterated analogues was described, resulting in minor changes in retention times [56].

These phenomena may explain the lower recovery of the deuterated internal standard and, therefore, the higher recovery of the test substance observed in this study. However, the best recoveries of the test substances were obtained when deuterated analogs were used for the analysis. Moreover, optimal chromatographic separation of piperazine derivatives in order to eliminate the deleterious effect of the matrix on the method performance was very important. This allows for the reduction of co-eluting matrix components. Notwithstanding, the key role in partial or even complete error correction caused by matrix effects should be assigned to a properly selected internal standard [51,56]. The research conducted by Matuszewski [55] showed that the use of stable internal standards isotope-labeled effectively eliminates the relative susceptibility to the matrix effect [55].

### 4.2. Analytical Confirmation of the Detection of Piperazine Derivatives in Biological Material

When using LC-MS, it is important to develop the samples to avoid the ion suppression effect [57]. The isolation of the tested compounds should take place at the pH at which the analyte is non-ionized. Piperazine derivatives are basic compounds [30]. Benzylpiperazines are hydrophilic and as polar analytes elute at the beginning of the gradient using reverse phase chromatography [45,51]. Phenylpiperazines: mCPP and TFMPP are hydrophobic and weakly polar due to the phenyl and halogen groups in the chemical structure [9]. This results in a longer retention time for these compounds compared to the benzylpiperazine derivatives. In the research on the detection of piperazine derivatives, initial alkalization of the 1 M NaOH environment did not bring the expected results. During the quantification, suppression of deuterated analyte ions occurred, which was the reason for overestimated results. Sample preparation was modified by alkalizing the 3 M NaOH environment, which allowed for obtaining satisfactory, repeatable results of quantitative analysis. The review of literature data shows that the concentrations of piperazine derivatives in blood and urine are high enough that their presence can be analytically confirmed by the proposed method. In previous studies, BZP was identified in the serum in the concentration range of 260–585 ng/mL, TFMPP 24–60 ng/mL, and mCPP 54 ng/mL [6,23]. In turn, the reported concentrations of BZP and TFMPP detected in the urine of people with poisoning ranged from 5.21 μg/mL to 202.7 μg/mL and from 0.40 μg/mL to 20.66 μg/mL, respectively [31]. In the fatal case, after consuming about 20 mg of mCPP in the analysed urine sample, this compound was detected in a concentration of 15.0 ng/mL [19].

The presented method was designed to detect piperazine derivatives in unmetabolized form. However, if standards of metabolites are available, the method can also be adapted to their determination. The method can also be useful in distinguishing an abuse from a treatment. For example, mCPP belongs to designer drugs, and is also an active metabolite, e.g., the antidepressant drug trazodone. Using the analytical technique presented, the therapeutic consumption of trazodone can be distinguished from the recreational consumption of mCPP. The concept of this method can be adapted and applied to the determination of other compounds, e.g., from the group of synthetic cannabinoids or ketoarylamines. However, it is necessary to find and mark the standards of these substances in order to unambiguously identify them in the biological material. In some cases, modification in equipment can be required. In practice, this means defining new customized method.

The advantage of the method with the use of LC-MS presented in this article is the high sensitivity of the determination of piperazine derivatives without the need to derivatize the sample, which significantly facilitates the work compared to the previously developed methods based on GC-MS [8,48]. The ability to determine serum or urine samples in a short time allows the current health status of a patient with ongoing poisoning to be assessed, unlike the analysis of hair samples, which indicates a chronic process of taking piperazine derivatives [42,43]. Developed method is distinguished from other methods by the use of deuterated analogues as internal standards, which ensures repeatability of quantitative determinations [44,46,48,51]. Additionally, other studies using LC-MS or GC-MS were usually not targeted explicitly to compounds from the group of piperazine derivatives [9,42,46,47].

Among other techniques, selectively for BZP, an electrochemical, voltammetric method of analysis and microcrystalline tests using mercury chloride have been developed [58,59]. Philp et al. [60] developed a colour method for identifying piperazine derivatives using the NQS (1,2-naphthoquinone-4-sulfonate) reagent, and Waite et al. [61] used chemilumunescence detection using tris (2,2′-bipyridine) ruthenium (III) as a reagent for the detection of piperazine analogues [60,61]. However, these methods require analytical confirmation by another technique and the reagents used require stability and selectivity controls. Procedures using capillary electrophoresis for the simultaneous separation of amphetamine and piperazine compounds are also described; however, for toxicological purposes it is necessary to provide lower detection limits [62]. The method proposed in this article supports these requirements and can be used to confirm the presence of relatively low levels of piperazine derivatives.

When identifying piperazine derivatives in biological material, it is also important to take into account all circumstances of the poisoning event for the correct interpretation of the result. Some piperazine derivatives may exist in biological fluids as metabolites of therapeutic drugs. For example, the mCPP is an active metabolite of drugs such as: trazodone, nefazodone, etoperidone, enziprazole, mepiprazole [6,31]. MDBP is a metabolite of the drug fipexide that has been withdrawn from therapy due to side effects, including liver toxicity. Another piperazine derivative, MeOPP, is also a metabolite of some drugs, such as urapidil [31]. BZP and its derivatives have in the past been tested as antidepressants but without medical use due to amphetamine-like effects, though less intense [5,7]. The phenylpiperazine derivative, TFMPP, has been identified as the major metabolite of antrafenine, an analgesic and anti-inflammatory drug [41]. Additionally, the metabolism of piperazine derivatives may indicate a potential problem with the interaction of concurrently administered drugs metabolized with the use of cytochrome P450 [31]. Drugs that inhibit the metabolism of cytochrome P450 (e.g., selective serotonin reuptake inhibitors SSRI) can multiply the pharmacological and toxic effects of other ingested compounds [57]. For example, the co-administration of fluoxetine may result in a four-fold increase of mCPP levels in plasma [31]. In clinical toxicology, it is important to recognize or definitively rule out acute or chronic poisoning [57]. Therefore, the first step should be always to correctly identify the compounds of interest.

## 5. Conclusions

Piperazine derivatives belong to popular, commonly abused compounds from the group of designer drugs. These compounds are being searched by consumers for recreational use due to their psychoactive and hallucinogenic effects similar to those of MDMA and amphetamines. Even a single, accidental use can lead to dangerous poisoning or death. The article describes the method that ensures the identification of piperazine designer drugs. The use of deuterated analogs of BZP-D7, mCPP-D8 and TFMPP-D4 as preferred internal standards has been documented. Each test compound was identified based on the precursor ion, at least two product ions, and the retention time. This allows the provision of qualitative evidence for the presence of piperazine derivatives. Additionally, the use of deuterated analogs as internal standards ensures repeatability of quantitative determinations. However, the use of deuterated internal standards that differ in structure requires further research.

The advantage of the method is the short analysis time combined with the simple procedure of preparing biological samples. In addition, it is possible to detect piperazine derivatives in a wide range of concentrations, including very small at the level of single ng. Correct identification of piperazine derivatives in biological material is necessary in comprehensive toxicological diagnosis. The presented method can be useful in laboratories for performing routine analyses to confirm the contribution of piperazine derivatives to the occurrence of poisoning.

## Figures and Tables

**Table 1:** Multiple reaction monitoring (MRM) table in the LC-MS system used.

Compound	Molecular Formula	Molecular Weight	Precursor Ion (*m*/*z*)	Mass Transitions	Collision Energy (CE)	Retention Time (t_R_)
BPZ	C_11_H_16_N_2_	176.26	177.3	177.3 → 90.95	25	1.194
				177.3 → 64.95	47	
MDBP	C_12_H_16_N_2_O_2_	220.27	220.95	220.95 → 135.00	17	1.371
				220.95 → 76.9	43	
pFBP	C_11_H_15_FN_2_	194.25	195.00	195.00 → 108.90	21	1.537
				195.00 → 83.00	47	
mCPP	C_10_H_13_C_l_N_2_	196.68	197.05	197.05 → 153.95	20	6.157
				197.05 → 117.95	36	
TFMPP	C_11_H_13_F_3_N_2_	230.23	230.95	230.95 → 187.95	24	6.756
				230.95 → 118.10	41	
				230.95 → 44.00	21	
BZP-D7	C_11_H_9_D_7_N_2_	183.33	184.15	184.15 → 98.00	23	1.103
				184.15 → 69.9	49	
mCPP-D8	C_10_H_5_D_8_ClN_2_	204.76	205.10	205.10 → 158.00	22	6.128
				205.10 → 123.00	27	
TFMPP-D4	C_11_H_9_D_4_F_3_N_2_	234.27	235.10	235.10 → 189.95	24	6.746
				235.10 → 121.05	34	
				235.10 → 46.00	23	

**Table 2:** Validation parameters (linear range, regression equation, determination coefficient (R^2^) and analytical limits).

Analytes	Internal Standard	Linear Range (ng/mL)	Regression Equation	R^2^	Analytes LOD (ng/mL)	Analytes LOQ (ng/mL)
BPZ	BZP-D7	1–1000	y = 0.0088x − 0.0076	0.9998	0.6552	1.9656
	mCPP-D8		y = 0.011x + 0.0981	0.9992		
	TFMPP-D4		y = 0.0055x + 0.0611	0.9990		
MDBP	BZP-D7	25–1000	y = 0.0171x + 0.1962	0.9969	0.2073	0.6219
	mCPP-D8		y = 0.0232x + 0.9232	0.9996		
	TFMPP-D4		y = 0.0116x + 0.491	0.9999		
pFBP	BZP-D7	1–1000	y = 0.0143x − 0.0879	0.9989	0.0042	0.0126
	mCPP-D8		y = 0.0194x + 0.1366	0.9995		
	TFMPP-D4		y = 0.0097x + 0.0889	0.9992		
mCPP	BZP-D7	1–1000	y = 0.0099x − 0.207	0.9906	1.419	4.257
	mCPP-D8		y = 0.0138x − 0.0573	0.9999		
	TFMPP-D4		y = 0.0066x − 0.0553	0.9977		
TFMPP	BZP-D7	1–1000	y = 0.02x − 0.3316	0.9952	0.1476	0.4428
	mCPP-D8		y = 0.027x − 0.1536	0.9986		
	TFMPP-D4		y = 0.0135x − 0.0512	0.9976		

**Table 3:** Repeatability of the retention times, surface areas of piperazine derivatives during the day and between days, expressed as the coefficient of variation (CV).

Analytes	Level	Daily Accuracy for t_R_, *n* = 9 CV (%)	Daily Accuracy for AUC, *n* = 9 CV (%)	Accuracy between Days for t_R_ CV (%)	Accuracy between Days for AUC CV (%)
BPZ	LQC	0.11	0.98	1.03	7.79
	MQC	0.08	0.53	1.41	7.82
	HQC	0.27	0.91	1.53	7.64
MDBP	LQC	0.07	1.44	1.09	8.00
	MQC	0.10	0.80	1.32	9.90
	HQC	0.21	1.03	1.50	10.77
pFBP	LQC	0.34	2.47	2.16	3.48
	MQC	0.09	1.46	2.27	5.57
	HQC	0.17	1.42	2.30	6.11
mCPP	LQC	0.04	1.39	0.05	11.71
	MQC	0.11	1.23	0.11	11.39
	HQC	0.06	0.63	0.06	9.01
TFMPP	LQC	0.03	0.62	0.06	6.38
	MQC	0.12	1.28	0.14	4.71
	HQC	0.05	1.20	0.10	2.26

**Table 4:** Results obtained from the analysis of biological material (urine, serum).

**Analytes**			**Urine**			**Serum**	
		Average 100 ng	Standard Deviation	%CV	Average 100 ng	Standard Deviation	%CV
BPZ	**BZP-D7**	**117.68**	3.92	3.34	**113.55**	3.82	3.36
	mCPP-D8	165.59	2.68	1.62	164.44	3.67	2.23
	TFMPP-D4	223.49	2.02	0.90	215.90	1.44	0.67
MDBP	**BZP-D7**	**100.87**	0.35	0.35	**102.51**	5.61	5.48
	mCPP-D8	106.80	2.80	2.62	123.28	6.49	5.26
	TFMPP-D4	165.51	4.55	2.75	168.57	1.91	1.13
pFBP	**BZP-D7**	**98.76**	4.34	4.40	**116.88**	3.38	2.89
	mCPP-D8	120.57	3.65	3.03	149.75	2.80	1.87
	TFMPP-D4	162.37	3.76	2.32	196.16	1.99	1.02
mCPP	BZP-D7	93.84	0.84	0.90	100.81	2.54	2.52
	**mCPP-D8**	**101.98**	0.65	0.66	**114.25**	2.10	1.84
	TFMPP-D4	145.88	1.85	1.27	159.11	1.27	0.80
TFMPP	BZP-D7	67.00	2.06	3.08	71.84	1.70	2.37
	mCPP-D8	75.50	1.58	2.10	84.32	1.42	1.69
	**TFMPP-D4**	**97.64**	1.50	1.53	**106.76**	0.97	0.91

bold is required to highlight selected selected results. Text above the table explains that.

**Table 5:** List of MRM transitions.

Compound	Established Mass Transitions	Literature Data	References
BZP	177.3 → 90.95	177.11 → 91 and 65	[28]
	177.3 → 64.95	177.0 → 91.1 and 65.1	[44]
		177.1 → 91.1 and 65.1	[30]
		177.13 → 91.05 and 65.038	[46]
MDBP	220.95 → 135.00	221.0 → 135.1 and 76.9	[44]
	220.95 → 76.9	220.9 → 135.0 and 76.9	[47]
pFBP	195.00 → 108.90	195.2 → 109.1 and 83.2	[30]
	195.00 → 83.00		
mCPP	197.05 → 153.95	197.11 → 153.9 and 118	[28]
	197.05 → 117.95	197.0 → 154.1 and 118.1	[44]
		197.1 → 154.2 and 118.2	[30]
		197.08 → 154.04 and 119.07	[46]
		197.1 → 153.9	[43]
TFMPP	230.95 → 187.95	231.11 → 188 and 118	[28]
	230.95 → 118.10	231.0 → 188.2 and 118.1	[44]
	230.95 → 44.00	231.1 → 188.1 and 118.3	[30]
		231.11 → 188.06	[46]
		231.1 → 188 and 44.1	[43]
		231.1 → 188.0 and 119.1	[47]
BZP-D7	184.15 → 98.00	184.11 → 98.1 and 70.1	[28]
	184.15 → 69.9	184.3 → 98.2	[30]
mCPP-D8	205.10 → 158.00	205.4 → 158.2	[30]
	205.10 → 123.00		
TFMPP-D4	235.10 → 189.95	235.11 → 190 and 46.1	[28]
	235.10 → 121.05	235.4 → 190.2	[30]
	235.10 → 46.00		
